# Adverse childhood experiences on internet gaming disorder mediated through insomnia in Chinese young people

**DOI:** 10.3389/fpubh.2023.1283106

**Published:** 2023-11-22

**Authors:** Ningyuan Guo, Xue Weng, Sheng Zhi Zhao, Juan Zhang, Man Ping Wang, Li Li, Lin Wang

**Affiliations:** ^1^School of Nursing, Shanghai Jiao Tong University, Shanghai, China; ^2^Institute of Advanced Studies in Humanities and Social Sciences, Beijing Normal University, Zhuhai, China; ^3^School of Nursing, The University of Hong Kong, Hong Kong, Hong Kong SAR, China; ^4^The International Peace Maternity and Child Health Hospital, Shanghai Jiao Tong University School of Medicine, Shanghai, China; ^5^Shanghai Mental Health Center, Shanghai Jiao Tong University School of Medicine, Shanghai, China

**Keywords:** adverse childhood experiences, insomnia, internet gaming disorder, mediation, life course

## Abstract

**Background:**

Adverse childhood experiences (ACEs) have been associated with addictions such as substance use disorders. Few have examined ACEs on internet gaming disorder (IGD) as a newly established behavioral addiction, and the potential mediating role of insomnia remains unclear. We examined the associations between ACE number and types, IGD, and insomnia.

**Methods:**

Participants included 1, 231 Chinese university students (54.5% male; 56.9% aged 18–20 years) who had played internet games at least once in the previous month. ACEs were measured using the 10-item ACE questionnaire (yes/no). Symptoms of insomnia and IGD were measured using the Insomnia Severity Index and the 9-item Internet Gaming Disorder Scale–Short-Form, respectively. Multivariable regressions examined the associations, adjusting for sex, age, maternal and paternal educational attainment, monthly household income, smoking, and alcohol drinking. The mediating role of insomnia symptoms was explored.

**Results:**

The prevalence of ACEs≥1 was 40.0%. Childhood verbal abuse was the most prevalent (17.4%), followed by exposure to domestic violence (17.1%) and childhood physical abuse (15.5%). More ACE numbers showed an association with IGD symptoms (adjusted OR = 1.11, 95% CI 1.04, 1.17). Specifically, IGD symptoms were observed for childhood physical neglect, emotional neglect, sexual abuse, parental divorce or separation, and household substance abuse. Insomnia symptoms mediated the associations of ACE number and types with IGD symptoms (proportion of total effect mediated range 0.23–0.89).

**Conclusion:**

The number and specific types of ACEs showed associations with IGD mediated through insomnia. Screening of ACEs is recommended in future studies on IGD. Longitudinal data are warranted to determine the causality of the observed associations.

## Introduction

Internet gaming disorder (IGD) refers to a new type of mental disorder due to addictive behavior that has been included in ICD-11 by WHO since 2019 (1). IGD has been prevalent in young people worldwide, and the prevalence rate is consistently higher in East Asia compared to other regions (2, 3). Adverse health outcomes associated with IGD, including physical and mental health problems, poor academic and work performance, and low levels of social skills and social problems have been increasingly reported (4, 5). However, the risk factors and underlying mechanisms of IGD remain mixed.

Billieux's integrative pathway model proposed that factors in adulthood, such as personal traits, emotional instability, and depression and anxiety symptoms, can increase the risks for IGD (6). This theoretical model has been widely used and supported by many studies (7, 8). Nevertheless, adult mental disorders can be traced back to early life (9). The more comprehensive Interaction of Person-Affect-Cognition-Execution (I-PACE) model of IGD posited that early childhood experiences were part of an individual's core characteristics, together with individual personality, cognition, and psychopathology in adulthood (10).

Adverse childhood experiences (ACEs) include childhood neglect (physical/emotional), childhood abuse (physical/abuse), and household dysfunction that an individual experiences before the age of 18 years (11). Established evidence supported that ACEs showed moderate to strong associations with substance use disorders (12) and addictive symptoms, which were similar to IGD. Some reported that ACE number was linearly associated with IGD (13, 14). Such findings provided an understanding of the cumulative health impact of ACEs, with the assumption of equal weighting for each ACE type (15, 16). For example, the combination of household mental illness and parental divorce or separation (ACE number 2) is treated as the same as childhood physical and sexual abuse (also an ACE number 2), which is unlikely to be the case (15, 16). Evidence has suggested that the impact of each ACE type on health outcomes could vary in the real world (17). Compared with the other types, childhood emotional neglect was found to have a stronger association with depression (18). Household substance use, physical abuse, and sexual abuse, in particular, were the strongest predictors of substance use disorders (19). Though depression and substance use disorders often co-occur with IGD (20), less is known about the different associations between each ACE type and IGD.

Insomnia is also a prevalent public health concern, and young people with insomnia symptoms were more likely to report higher IGD risks (21). A longitudinal study showed that insomnia symptoms predicted longer gaming time, which was an established predictor of IGD (22). Insomnia symptoms may lead to daytime tiredness and few physical activities (23), thus engaging in sedentary activities such as internet gaming. Internet gaming may also act as a maladaptive coping strategy for negative moods and poor emotional adjustment induced by insomnia symptoms (24). In addition, individuals with ACEs consistently reported higher levels of insomnia symptoms, probably due to circadian dysregulation, increased neuron activity in the brain, improper sleep habits, and other pathways (25). These findings suggested that insomnia symptoms could be a possible mechanism linking ACEs with IGD. As sleep behaviors can be modifiable targets of health management, investigations of the potential mediating role of insomnia on the association of ACEs with IGD could inform early preventions and interventions for IGD in young people.

China has the biggest gaming market worldwide (26). The number of internet gaming users reached 552 million in June 2022 (27), of whom 77 million (14%) might be at risk for IGD (28). The prevalence appeared to be higher in Chinese young people who have been growing up with digital devices and in a pandemic when outdoor activities were restricted and screen time increased (29). We aimed to report the prevalence of ACEs in Chinese young people and examine the associations of ACE number and types with IGD symptoms. The mediating role of insomnia symptoms was explored.

## Methods

### Participants and procedure

We conducted a cross-sectional study of university students in China from December 2022 to January 2023. Inclusion criteria are as follows: age≥18 years, full-time student, and had played internet gaming at least once in the previous month. Invitations to the online survey were distributed *via* WeChat, one of the most popular social networking sites in China. Interested students were screened for eligibility, and eligible individuals completed the online survey. Participants earned a cash incentive of CNY10 (equivalent to USD1.49) for completion. The survey was anonymous and programmed to allow single completions per device to prevent duplicate submissions.

### Measurements

#### Adverse childhood experiences

ACEs were measured using the 10-item ACE questionnaire developed by the Centers for Disease Control and Prevention (CDC), US (11). Childhood neglect (emotional and physical), childhood abuse (emotional, physical, and sexual), and household dysfunction (incarcerated household member, parental divorce or separation, exposure to domestic violence, household substance abuse, and household mental illness) were all asked about by age 18, with a yes/no response to each item (11). Responses were summed to generate a cumulative ACE score ranging from 0 to 10. The questionnaire is one of the most used measurements of ACEs worldwide and has shown reliability and validity in the Chinese population (30). Cronbach's α was 0.86 in the present study.

#### Internet gaming disorder

IGD symptoms were measured using the 9-item Internet Gaming Disorder Scale–Short-Form (IGDS-SF9) on nine diagnostic criteria of IGD in DSM-5, including preoccupation, withdrawal, tolerance, relapse, loss of interest, continued and excessive use, deception, mood modification, losing interpersonal relationships, and work and educational opportunities (31). Each item was on a 5-point Likert scale from 1 = never to 5 = very often, with a higher total score (range 9–45), indicating higher IGD symptom severity (31). The Chinese version of IGDS-SF9 has been validated (32). The score of 32 has been evident as the optimal cutoff point for screening IGD symptoms in the Chinese population (33). Cronbach's α was 0.95 in the present study.

#### Insomnia

Insomnia symptoms were measured using the seven-item Insomnia Severity Index (ISI) on dimensions of the severity of sleep onset, sleep maintenance, early morning awakening problems, sleep dissatisfaction, interference of sleep difficulties with daytime functioning, noticeability of sleep problems by others, and distress caused by the sleep difficulties that occurred in the previous month (34). Each item was on a 5-point Likert scale from 0 = no problem to 4 = very severe problem, with a higher total score (range 0–28) indicating greater insomnia severity (34). The Chinese version of ISI has been validated (35). A score of 10 has been recommended to use community-based samples for screening insomnia symptoms (36). Cronbach's α was 0.90 in the present study.

#### Covariates

Covariates included sex, age, maternal and paternal educational attainment, and monthly household income. Smoking and alcohol drinking were also included due to their co-occurrence with IGD (37) and insomnia (38).

### Statistical analysis

We checked the distributions of all variables independently, with a skewness value of |2.0| and a kurtosis value of |7.0|, indicating normality (39). The mean score of the number and proportion of different types of ACEs were presented. We first examined the association of ACE number with symptoms of IGD and insomnia using bivariate and multivariable logistic regression analyses adjusting for sex, age, maternal and paternal educational attainment, monthly household income, smoking, and alcohol drinking. Then, we differentiated the associations of different types of ACEs with symptoms of IGD and insomnia. The variance inflation factor (VIF) was calculated for each of the multivariable regression models, with values ranging from 1.32 to 1.35, indicating the low possibility of multicollinearity (40). For any statistically significant association, direct association and indirect association through insomnia symptoms (i.e., the potential mediator) were decomposed (41) using the “mediate” command in STATA. All analyses were conducted using STATA version/MP 18 (StataCorp., TX, USA). A *P* < 0.05 was considered statistically significant.

### Ethics

The study procedures were carried out in accordance with the Declaration of Helsinki. The Institutional Review Board of the Shanghai Jiao Tong University School of Medicine approved the study. Implied consent to participate was indicated when participants provided responses to survey items.

## Results

Table 1 shows that of the 1, 231 participants, 54.5% were male and 56.9% were aged 18–20 years. The mean (SD) number of ACEs was 1.3 (2.3). The proportions of screening positives for symptoms of IGD and insomnia were 28.7 and 43.2%, respectively.

**Table 1:** Characteristics of participants (*N* = 1,231).

	***n* (%)/ mean ±SD**
**Sex**	
Female	560 (45.5)
Male	671 (54.5)
**Age (years)**	
18	197 (16.0)
19	220 (17.9)
20	283 (23.0)
21	208 (16.9)
22	169 (13.7)
≥23	154 (12.5)
**Maternal educational attainment**	
≤ Primary school	129 (10.5)
Middle school	284 (23.1)
High school or equivalent	415 (33.7)
≥Undergraduate degree	403 (32.7)
**Paternal educational attainment**	
≤ Primary school	122 (9.9)
Middle school	320 (26.0)
High school or equivalent	431 (35.0)
≥Undergraduate degree	358 (29.1)
**Monthly household income (RMB, 1 USD**≈**7.0 RMB)**	
< 5000	189 (15.4)
5000–9999	396 (32.2)
10000–14999	276 (22.4)
15000–19999	180 (14.6)
≥20000	190 (15.4)
ACE number (range 0–10)	1.3 ± 2.3
Any ACE (ACE number>1)	492 (40.0%)
**Insomnia symptoms**	
ISI < 10	699 (56.8)
ISI≥10	532 (43.2)
**IGD symptoms (score, range 9–45)**	
IGDS-SF9 < 32	828 (71.3)
IGDS-SF9≥32	353 (28.7)

ACEs, adverse childhood experiences; ISI, Insomnia Severity Index; IGD9-SF9, 9-item Internet Gaming Disorder Scale–Short-Form.

**Figure 1:**
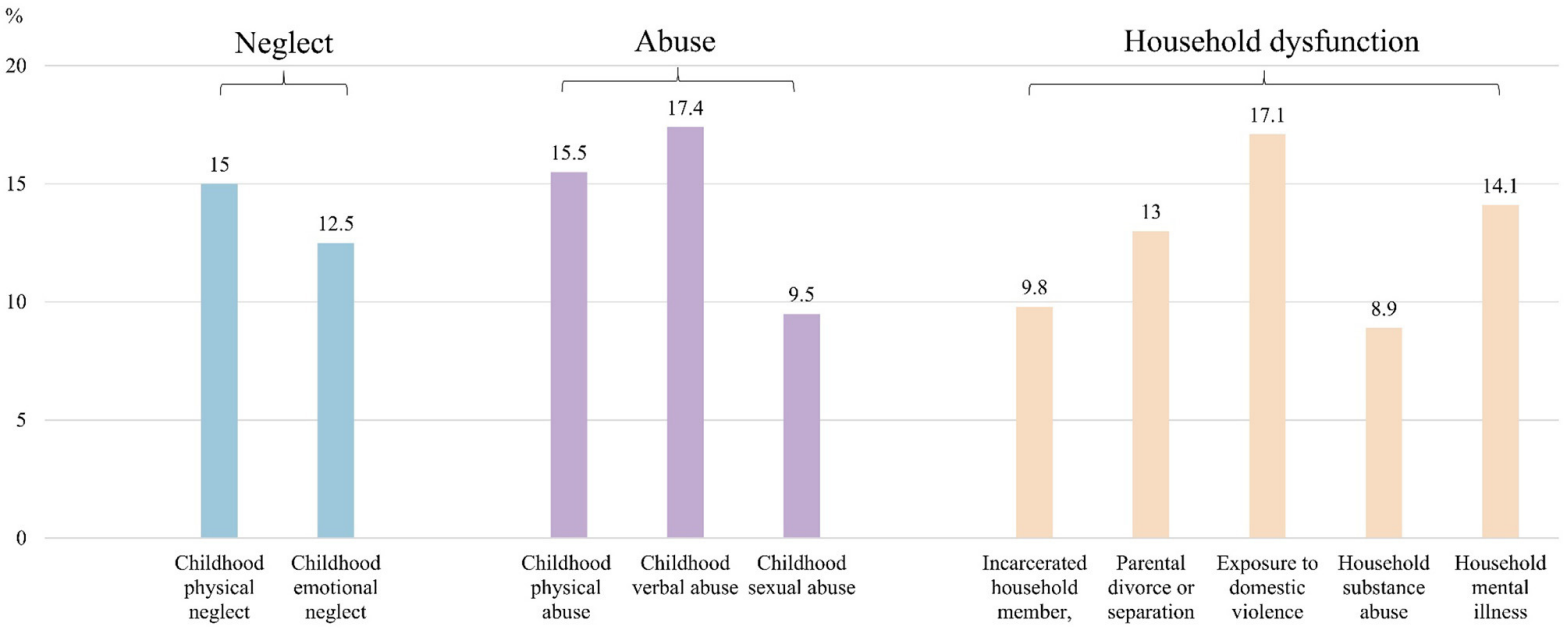
Prevalence of different types of adverse childhood experiences (*N* = 1,231).

Figure 1 shows that the most prevalent type of ACEs was childhood verbal abuse (214 of 1,231, 17.4%), followed by exposure to domestic violence (210 of 1,231, 17.1%) and childhood physical abuse (191 of 1,231, 15.5%).

Table 2 shows that for every increase in ACE number, the odds of IGD symptoms increased by 0.17 (OR = 1.17, 95% CI 1.11, 1.23). The association remained significant (adjusted OR = 1.11, 95% CI 1.04, 1.17) after adjusting for sex, age, maternal and paternal educational attainment, monthly household income, smoking, and alcohol drinking. Specifically, higher IGD symptoms were observed for childhood physical neglect (adjusted OR = 4.41, 95% CI 3.02, 6.44), childhood emotional neglect (adjusted OR = 1.54, 95% CI 1.03, 2.30), childhood sexual abuse (adjusted OR = 1.81, 95% CI 1.17, 2.82), parental divorce or separation (adjusted OR = 1.94, 95% CI 1.31, 2.85), and household substance abuse (adjusted OR = 2.39, 95% CI 1.50, 3.79). In addition, each type of ACE was associated with insomnia symptoms (all *P*s < 0.05). The highest magnitude of the association with insomnia symptoms was observed for childhood physical neglect (adjusted OR = 3.85, 95% CI 2.62, 5.65), followed by household substance use (adjusted OR = 3.81, 95% CI 2.29, 6.36) and mental illness (adjusted OR = 3.80, 95% CI 2.53, 5.67).

**Table 2:** Associations of adverse childhood experiences with symptoms of internet gaming disorder and insomnia (*N* = 1,231).

	**IGDS-SF9**≥**32**		**ISI**≥**10**	
	**Crude OR (95% CI)**	**Adjusted OR (95% CI)** ^a^	**Crude OR (95% CI)**	**Adjusted OR (95% CI)** ^a^
ACEs number (range 0–10)	1.17 (1.11, 1.23)^***^	1.11 (1.04, 1.17)^**^	1.37 (1.29, 1.46)^***^	1.27 (1.20, 1.37)^***^
**ACEs-neglect**				
Physical neglect	5.67 (4.07, 7.89)^***^	4.41 (3.02, 6.44)^***^	5.32 (3.71, 7.63)^***^	3.85 (2.62, 5.65)^***^
Emotional neglect	1.94 (1.37, 2.75)^***^	1.54 (1.03, 2.30)^*^	3.37 (2.35, 4.84)^***^	2.94 (2.00, 4.32)^***^
**ACEs-abuse**				
Physical abuse	1.13 (0.81, 1.59)	1.00 (0.68, 1.47)	1.63 (1.19, 2.22)^**^	1.49 (1.07, 2.08)^*^
Verbal abuse	1.07 (0.78, 1.48)	0.88 (0.61, 1.27)	3.09 (2.27, 4.21)^***^	2.53 (1.82, 3.53)^***^
Sexual abuse	2.43 (1.65, 3.58)^***^	1.81 (1.17, 2.82)^**^	3.78 (2.49, 5.76)^***^	2.95 (1.89, 4.61)^***^
**ACEs-household dysfunction**				
Incarceration	2.03 (1.38, 2.97)^***^	1.47 (0.94, 2.31)	5.34 (3.42, 8.33)^***^	3.53 (2.19, 5.69)^***^
Divorce or separation	2.44 (1.74, 3.43)^***^	1.94 (1.31, 2.85)^**^	2.62 (1.86, 3.70)^***^	1.96 (1.35, 2.84)^***^
Domestic violence	1.11 (0.80, 1.53)	0.96 (0.66, 1.39)	2.83 (2.08, 3.85)^***^	2.32 (1.67, 3.23)^***^
Substance abuse	4.10 (2.74, 6.14)^***^	2.39 (1.50, 3.79)^***^	6.02 (3.71, 9.75)^***^	3.81 (2.29, 6.36)^***^
Mental illness	2.25 (1.62, 3.12)^***^	1.48 (0.99, 2.21)	5.75 (3.95, 8.39)^***^	3.80 (2.53, 5.67)^***^

* P < 0.05;

** P < 0.01;

*** P < 0.001; ISI, Insomnia Severity Index; IGD9-SF9, 9-item Internet Gaming Disorder Scale–Short-Form.

a Adjusted for sex, age, maternal and paternal educational attainment, monthly household income, smoking, and alcohol drinking.

Table 3 shows that the association of ACE number with IGD symptoms was fully mediated through insomnia symptoms (indirect association: adjusted ORs range 1.06–2.13, all *P*s < 0.001; proportion of total association mediated: 0.85–0.89). Insomnia symptoms also fully mediated the association of childhood emotional neglect (indirect association: adjusted OR = 1.36, 95% CI 1.14, 1.63; proportion of total association mediated: 0.93), childhood sexual abuse (indirect association: adjusted OR = 1.33, 95% CI 1.07, 1.64; proportion of total association mediated: 0.64), parental divorce or separation (indirect association: adjusted OR = 1.26, 95% CI 1.09, 1.47; proportion of total association mediated: 0.53), and household substance abuse (indirect association: adjusted OR = 1.55, 95% CI 1.18, 2.03; proportion of total association mediated: 0.67) with IGD symptoms. The partial mediating role of insomnia symptoms was observed for IGD symptoms associated with childhood physical neglect (indirect association: adjusted OR = 1.30, 95% CI 1.07, 1.58; proportion of total association mediated: 0.23).

**Table 3:** Decomposition of total associations of adverse childhood experiences on internet gaming disorder symptoms mediated through insomnia symptoms.

	**IGD9-SF9** ≥**32, adjusted OR (95% CI)**^**a**^			
	**Total association**	**Indirect association**	**Direct association**	**Proportion of total association mediated**
**ACEs number (reference: 0)**				
1	1.07 (1.00, 1.13)^*^	1.06 (1.03, 1.08)^***^	1.01 (0.95, 1.07)	0.85
2	1.15 (1.02, 1.29)^*^	1.12 (1.07, 1.18)^***^	1.02 (0.91, 1.15)	0.86
3	1.25 (1.06, 1.47)^**^	1.21 (1.12, 1.31)^***^	1.03 (0.87, 1.23)	0.87
4	1.36 (1.11, 1.67)^**^	1.30 (1.16, 1.47)^***^	1.04 (0.83, 1.31)	0.87
5	1.49 (1.16, 1.92)^**^	1.42 (1.21, 1.66)^***^	1.05 (0.80, 1.39)	0.88
6	1.64 (1.22, 2.20)^**^	1.54 (1.25, 1.89)^***^	1.07 (0.88, 1.48)	0.88
7	1.81 (1.29, 2.53)^**^	1.67 (1.30, 2.16)^***^	1.08 (0.74, 1.57)	0.88
8	1.99 (1.35, 2.92)^***^	1.82 (1.34, 2.46)^***^	1.09 (0.72, 1.66)	0.88
9	2.18 (1.41, 3.36)^***^	1.97 (1.39, 2.80)^***^	1.11 (0.70, 1.75)	0.88
10	2.39 (1.47, 3.87)^***^	2.13 (1.43, 3.17)^***^	1.12 (0.68, 1.84)	0.89
**ACEs-neglect**				
Physical neglect (reference: no)	3.38 (2.46, 4.63)^***^	1.30 (1.07, 1.58)^**^	2.60 (1.80, 3.76)^***^	0.23
Emotional neglect (reference: no)	1.40 (1.02, 1.91)^*^	1.36 (1.14, 1.63)^**^	1.03 (0.73, 1.43)	0.93
**ACEs-abuse**				
Sexual abuse (reference: no)	1.58 (1.11, 2.25)^*^	1.33 (1.07, 1.64)^**^	1.19 (0.79, 1.79)	0.64
ACEs-household dysfunction				
Divorce or separation (reference: no)	1.59 (1.16, 2.17)^**^	1.26 (1.09, 1.47)^**^	1.26 (0.93, 1.70)	0.53
Substance abuse (reference: no)	1.97 (1.34, 2.89)^**^	1.55 (1.18, 2.03)^**^	1.27 (0.83, 1.96)	0.67

* P < 0.05;

** P < 0.01;

*** P < 0.001; IGD9-SF9, 9-item Internet Gaming Disorder Scale–Short-Form.

a Adjusted for sex, age, maternal and paternal educational attainment, monthly household income, smoking, and alcohol drinking.

## Discussion

Our study showed that 40.0% of participants reported ACEs at least once before the age of 18 years. Specifically, childhood verbal abuse was the most common type of ACEs (17.4%), followed by exposure to domestic violence (17.1%) and childhood physical neglect (15.0%). ACE number and specific types (i.e., childhood physical neglect, emotional neglect, sexual abuse, parental divorce or separation, and household substance abuse) were associated with higher IGD symptoms through the mediating role of higher insomnia symptoms.

Much of ACE research studies have focused on populations in Western countries, particularly in the US, as the original ACE research was conducted by Kaiser Permanente and the US CDC (11). Our timely results added evidence of the prevalence of ACEs in Chinese young people. The proportion of participants reporting ACEs≥1 (40.0%) was comparable to that in 51, 945 adults in the World Mental Health Survey (38.3%) (42), while the proportion was lower than those reported in young adults in Brazil (74.4%) (43) and the US (61.6%) (44); notably, direct comparison may not be feasible as different measurements of ACEs were used. A meta-analysis showed that ACEs in Asia were less prevalent than in North and South America (45). This might be due to collectivist values or higher child protective policies in Asian countries, such as China (46). However, it should be noted that the cultural values in Asia might prevent victims of ACEs from reporting their experiences *via* a self-report survey. Specifically, childhood verbal abuse and physical abuse were the most common among all ACE types. This might be explained by the parenting styles of Chinese parents, which are mostly characterized by strictness (verbal scolding and physical punishment) (47). Exposure to domestic violence (17.1%) was the most common household dysfunction, and such a high prevalence called for studies, in particular, on intimate partner violence, which was understudied in China (48).

The association of ACE number with higher odds of IGD symptoms was in line with studies conducted in South Korea (13), Canada (14), and Poland (49). Prolonged stress induced by higher numbers of ACEs may activate hypothalamic–pituitary–adrenal (HPA) axis suppression (50). Such dysfunction has been shown in individuals with IGD (51), implying a neuroendocrine pathway-underlying mechanism through the cumulative association of ACE with IGD. ACEs as traumatic events may predispose individuals to higher risks for dissociative disorders, showing the tendency to escape from reality (49). As a substitute for reality, the frequency and duration of internet gaming may increase in young people, which were established risk factors for IGD. Another explanation can be due to the adverse impacts of ACEs on the development of brain circuits that regulate emotion and motivation, including neuroplasticity and neurogenesis, as evident by studies using animal models and population data (52). Such vulnerability to regulation in emotion and motivation has been suggested as the biological mechanism of addictions such as IGD (52).

Our study provided novel results by disentangling the associations of various ACE types with IGD symptoms. Specifically, childhood physical neglect was strongly associated with IGD symptoms. An association was also observed for childhood emotional neglect, with a smaller association magnitude. Emotional neglect may represent subjective perceptions of unmet emotional needs of a child, such as support, affection, and love, while physical neglect is characterized by an objective lack of nurturing and protection for the child. Previous research showed that non-supervision and non-discipline were associated with IGD (53). Higher IGD symptoms were also observed for individuals with sexual abuse. The Compounded Convergence of Mechanisms Model of Child Sexual Abuse posits that traumatic sexualization and insecure attachment are unique pathways to childhood sexual abuse, which can lead to addictions such as IGD (54). Previous studies have consistently reported concurrent familiar factors for IGD, as single-parent families, a broken household context, and a positive parental attitude toward child/adolescent substance use were associated with higher risks for IGD (4, 55). Our study complemented these results by demonstrating the association between household dysfunction in early life and IGD. We showed that household substance abuse and parental divorce or separation in the household were associated with higher IGD symptoms. Substance abuse by family members, in particular parents, has shown transgenerational effects through additive genetic variance and unhealthy lifestyles (56). Given the potential similarities between substance abuse and IGD (57), substance abuse by family members may increase the risk of IGD. The association between divorce or separation and IGD might be attributable to higher IGD risks induced by physical and emotional neglect (4). Future studies on the interrelations between childhood physical and emotional neglect and divorce or separation in the household are warranted to elucidate the mechanism of early life familiar factors for IGD.

All of the observed associations of ACE number/type with IGD symptoms were mediated through insomnia symptoms. Evidence from longitudinal studies, including our previous ecological momentary study, has supported the predicting role of insomnia symptoms on gaming time as established risk factors for IGD (21, 22). Insomnia symptoms in young adults with more ACEs could be explained by the circadian rhythm disorders caused by the ACE-induced HPA axis dysfunction in a dose-response relation (50). The highest proportion of total association mediated was observed for childhood emotional neglect compared to those of other types. Researchers have suggested that childhood emotional neglect could be a distinct type of ACE characterized by the absence or omission of emotional or psychological support compared to other types, such as childhood emotional and physical abuse, which might be more active acts of commission (58). Thereby, childhood emotional neglect may uniquely contribute to insomnia symptoms by impairing cognitive regulation of emotions and perceived stress. Young adults with childhood emotional neglect were more likely to have higher levels of loneliness and social isolation, which may increase the risk for insomnia symptoms (59). Nevertheless, more empirical studies were warranted to explore the underlying cognitive and psychosocial mechanisms linking childhood emotional neglect and insomnia symptoms.

Notably, no conclusions can be drawn in the direction of causality in the present cross-sectional study. The reverse direction, that IGD might lead to ACEs, can be possible and can be explored using a prospective approach in children and adolescents. IGD was associated with behavioral disorders, such as attention deficit hyperactivity disorder, oppositional defiant disorder, and conduct disorder (60). Behavioral disorders may induce conflicts in school and family that are adversities in childhood (61). Insomnia symptoms may mediate the association of IGD with ACEs, as IGD has shown an association with insomnia symptoms that often co-occur with ACEs in children/adolescents (62, 63). These findings, together with the results of the present study, suggest bidirectional relations among ACEs, insomnia, and IGD, which can be confirmed using a prospective approach in young people aged <18 years and a retrospective approach in adults.

This study has limitations. First, the convenience sampling method was subjected to volunteer bias, thereby making the representativeness of our results unclear. Large-scale population-based studies are needed to increase generalizability. Second, the cross-sectional data restricted causal inference on the associations between ACEs, insomnia, and IGD. Reverse direction in the association that ACEs induce IGD through insomnia can be possible, as well as bidirectional associations between ACEs, insomnia, and IGD. Future longitudinal observational and intervention studies are needed to infer causality. Third, retrospective measurements of ACEs were mostly used in adult studies (64, 65). However, such a method can be subjected to recall bias, particularly for less objective ACE types such as neglect. Notably, ACEs can be prospectively measured in individuals < 18 years old using repeated interviews, observations, and records (64, 65). Prospective and retrospective ACEs have shown low to moderate agreement and differential associations with health outcomes (66). Hence, future studies were suggested to compare prospective and retrospective associations of ACEs with insomnia and IGD. Self-reported data on insomnia and IGD can be further validated using objective measurements or clinical diagnoses of sleep and gaming behaviors.

Our results had potential implications for future research. The prevalence of ACEs at least once before the age of 18 years was 40% in a large sample of 1, 231 Chinese university students, with the most prevalent types being childhood verbal abuse, exposure to domestic violence, and childhood physical neglect. These findings, generated from a retrospective method, warranted prospective ACE surveillance studies in children/adolescents aged ≤ 18 years to confirm the prevalence of ACEs in Chinese young people. University-based screening programs for identifying students with higher ACEs can be developed and thereby provide specific psychological support. Health concerns about IGD as a new type of behavioral addiction have been increasing worldwide, particularly in Asian countries such as China. Higher IGD symptom severity observed with ACE number and specific type called for longitudinal studies to infer the causality. More observational and interventional research on the associations of ACE number and specific types with IGD is needed to confirm whether ACEs can be a risk factor for IGD. Our results highlighted the importance of training in positive parenting techniques, which have shown promise in reducing ACEs before the age of 18 and IGD in adulthood. The mediating role of insomnia symptom severity, if further confirmed using longitudinal data, warrants sleep interventions, such as effective cognitive behavioral therapy for insomnia (CBT-I), preventing IGD, and mitigating the adverse impacts of ACEs in young people.

## Conclusion

The number and specific types of ACEs showed associations with IGD symptoms through the mediating role of insomnia symptoms. Screening of ACEs is recommended in future studies on IGD. Longitudinal data are warranted to determine the causality of the observed associations.

## Data availability statement

The raw data supporting the conclusions of this article will be made available by the authors, without undue reservation.

## Ethics statement

The studies involving humans were approved by the Institutional Review Board of the Shanghai Jiao Tong University School of Medicine. The studies were conducted in accordance with the local legislation and institutional requirements. The participants provided their written informed consent to participate in this study.

## Author contributions

NG: Conceptualization, Data curation, Formal analysis, Funding acquisition, Investigation, Methodology, Project administration, Visualization, Writing—original draft, Writing—review & editing. XW: Conceptualization, Investigation, Methodology, Supervision, Validation, Visualization, Writing—review & editing. SZ: Methodology, Validation, Writing—review & editing. JZ: Writing—review & editing. MW: Supervision, Writing—review & editing. LL: Investigation, Methodology, Supervision, Validation, Writing—review & editing. LW: Conceptualization, Funding acquisition, Investigation, Methodology, Supervision, Validation, Visualization, Writing—review & editing.
